# Supplementary figures and images for: Domestic sewage dispersion scenarios as a subsidy to the design of urban sewage systems in the Lower Amazon River, Amapá, Brazil
Source: PeerJ. 2024 Feb 27;12:e16933. doi: 10.7717/peerj.16933 (PMC11636686; doi:10.7717/peerj.16933)

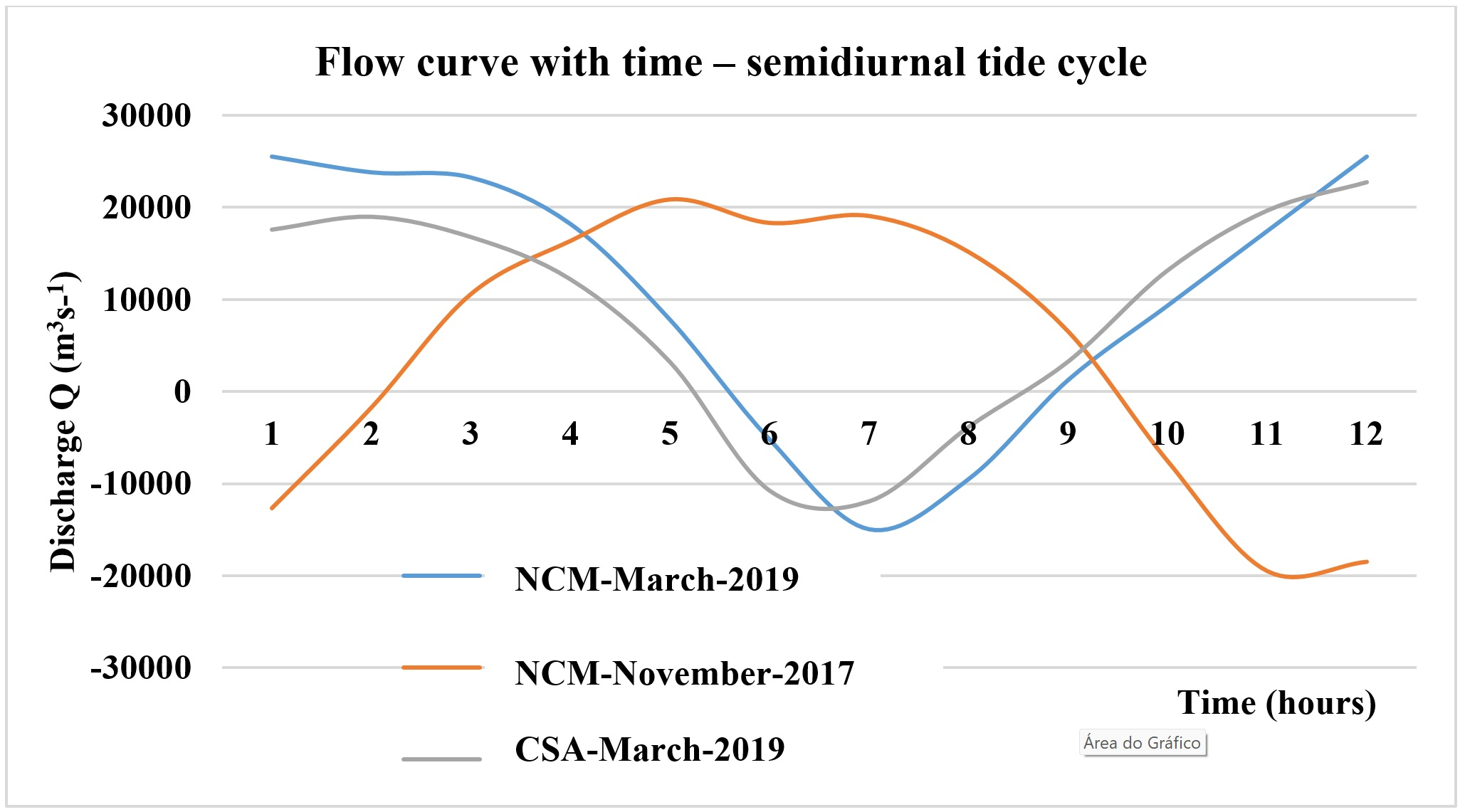

Supplement: Supplement S1 [file peerj-12-16933-s002.png]

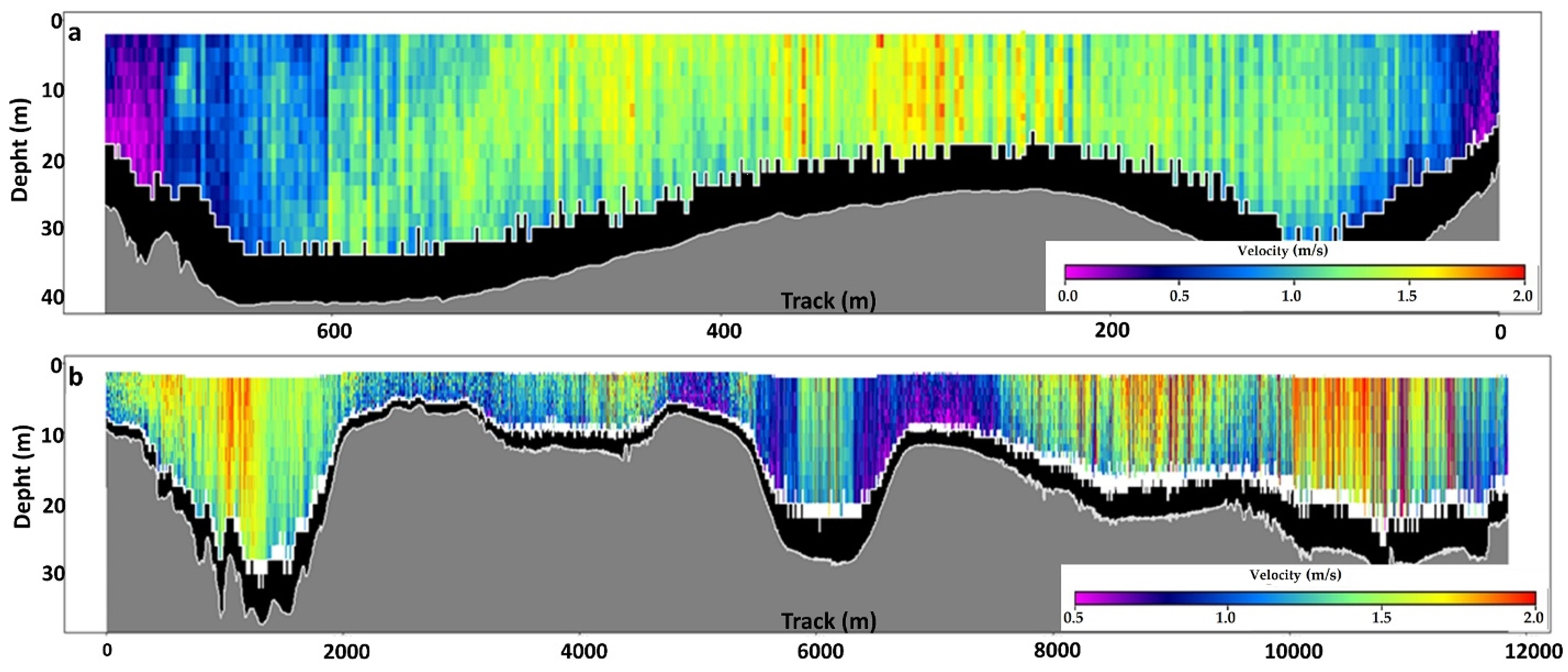

Supplement: Supplement S2 — Source credit: M. de Abreu et al. (2020), CC BY 4.0. [file peerj-12-16933-s003.png]

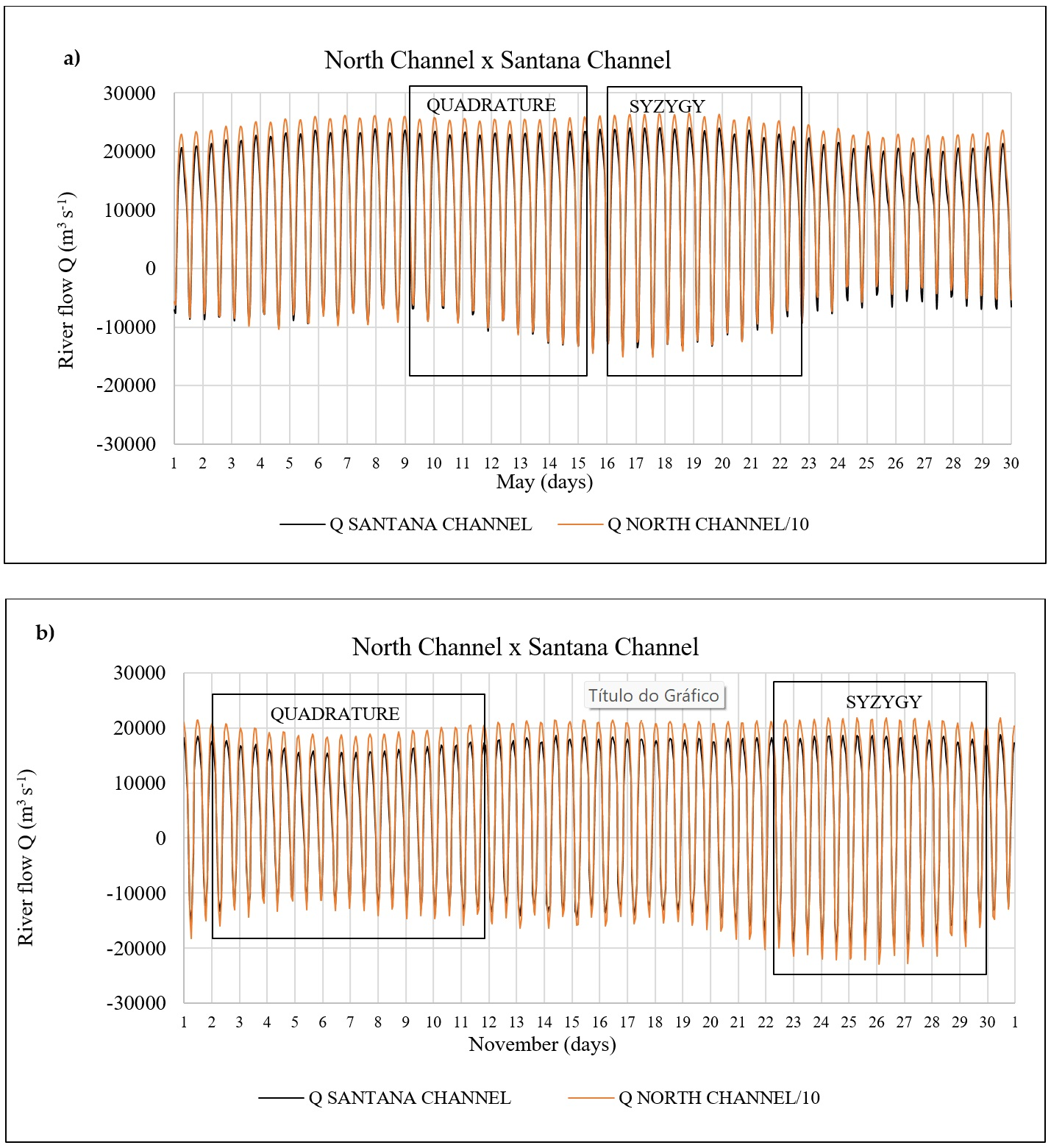

Supplement: Supplement S3 — Source credit: M. de Abreu et al. (2020), CC BY 4.0. [file peerj-12-16933-s004.png]

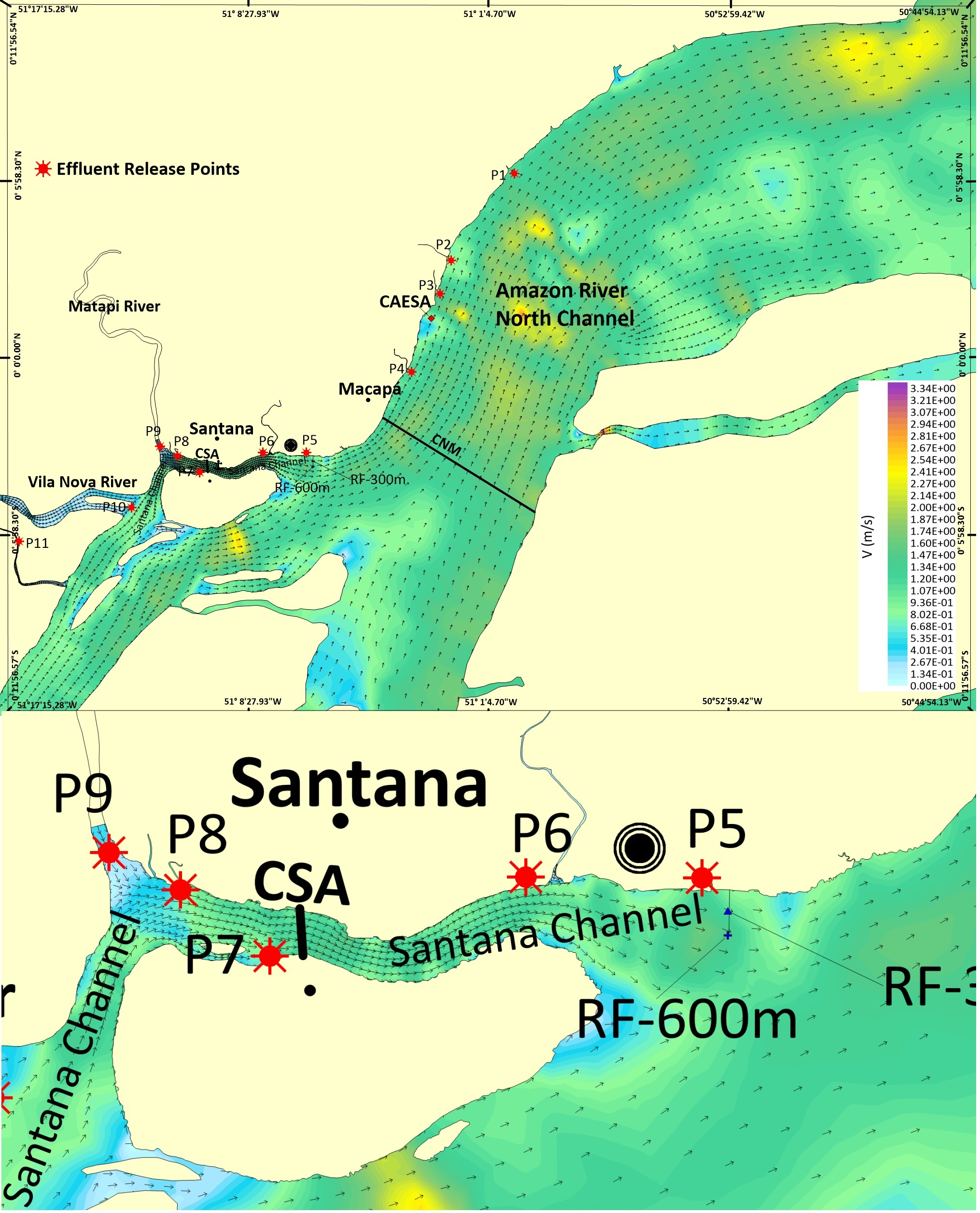

Supplement: Supplement S4 — (P1) Igarapé do Curiaú; (P2) Channel of Jandiá; (P3) Igarapé das Mulheres; (P4) Igarapé das Pedrinhas; (P5) Fazendinha Environmental Protection Area; (P6) Igarapé da Fortaleza; (P7) Island of Santana; (P8) Bairro do Elesbão –STN; (P9) Matapi River; (P10) Vila Nova River; (P11) Beija-Flor River. [file peerj-12-16933-s005.png]

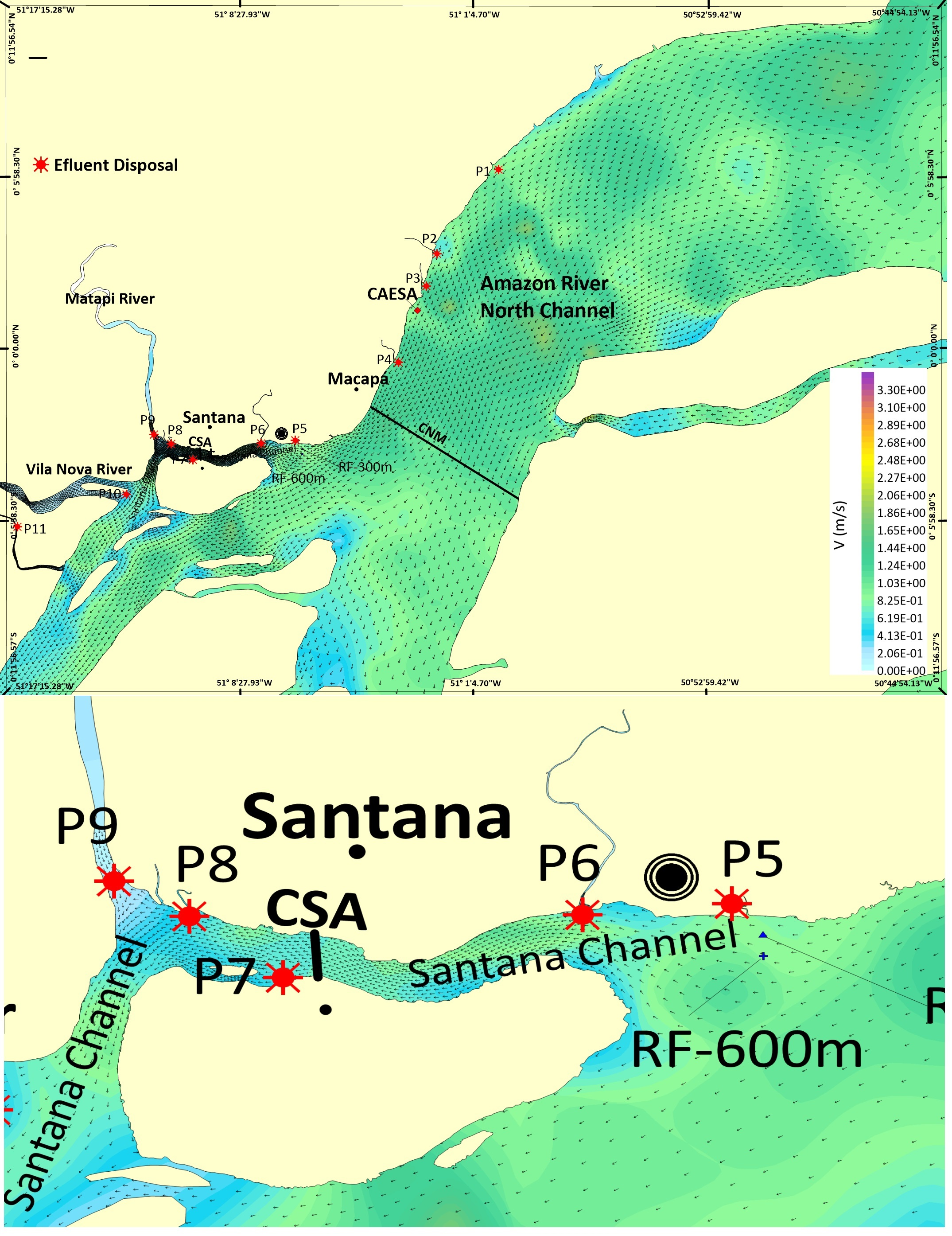

Supplement: Supplement S5 — (P1) Igarapé do Curiaú; (P2) Channel of Jandiá; (P3) Igarapé das Mulheres; (P4) Igarapé das Pedrinhas; (P5) Fazendinha Environmental Protection Area; (P6) Igarapé da Fortaleza; (P7) Island of Santana; (P8) Bairro do Elesbão –STN; (P9) Matapi River; (P10) Vila Nova River; (P11) Beija-Flor River. [file peerj-12-16933-s006.png]

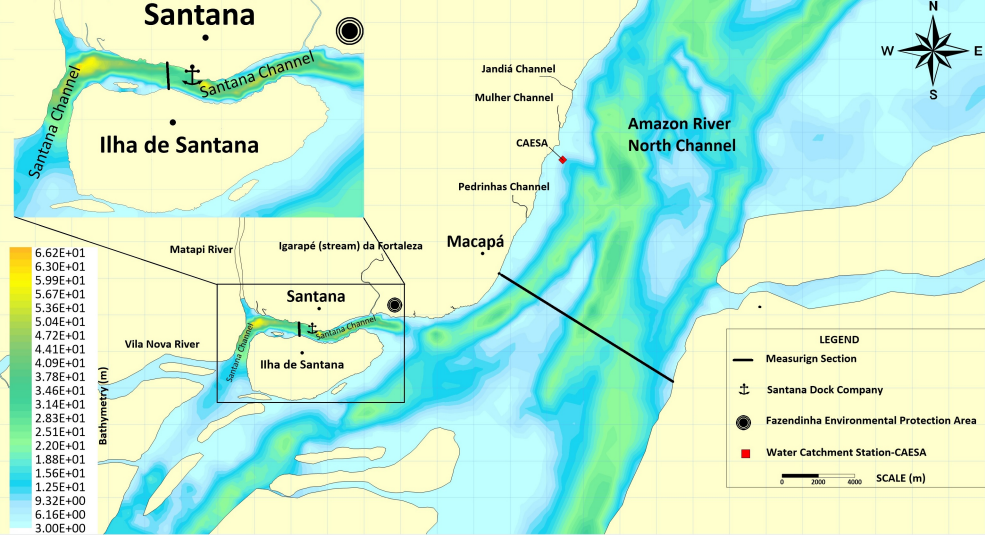

Supplement: Supplemental Information 7 — Bathymetric details, in meters, of the Santana Channel and North Channel of the Amazon River near the city of Macapá, related to the pollutant dispersion region. [file peerj-12-16933-s007.pdf]
